# The Effect of Flaxseed in Breast Cancer: A Literature Review

**DOI:** 10.3389/fnut.2018.00004

**Published:** 2018-02-07

**Authors:** Ana Calado, Pedro Miguel Neves, Teresa Santos, Paula Ravasco

**Affiliations:** ^1^Instituto de Ciências da Saúde, Universidade Católica Portuguesa, Lisbon, Portugal; ^2^Faculdade de Medicina da Universidade de Lisboa, Hospital Universitário de Santa Maria and Centro de Investigação Interdisciplinar em Saúde da Universidade Católica Portuguesa, Lisbon, Portugal; ^3^Faculdade de Motricidade Humana (FMH) (Projecto Aventura Social-Social Adventure Team), Universidade de Lisboa, Lisbon, Portugal; ^4^Instituto de Saúde Ambiental (ISAMB), Faculdade de Medicina, Universidade de Lisboa, Lisbon, Portugal; ^5^William James Center for Research, ISPA––Instituto Universitário, Lisbon, Portugal

**Keywords:** breast cancer, flaxseed, lignan, nutrition, omega-3

## Abstract

Breast cancer is one of the most common cancers and the second most responsible for cancer mortality worldwide. In 2014, in Portugal approximately 27,200 people died of cancer, of which 1,791 were women with breast cancer. Flaxseed has been one of the most studied foods, regarding possible relations to breast cancer, though mainly in experimental studies in animals, yet in few clinical trials. It is rich in omega-3 fatty acids, α-linolenic acid, lignan, and fibers. One of the main components of flaxseed is the lignans, of which 95% are made of the predominant secoisolariciresinol diglucoside (SDG). SDG is converted into enterolactone and enterodiol, both with antiestrogen activity and structurally similar to estrogen; they can bind to cell receptors, decreasing cell growth. Some studies have shown that the intake of omega-3 fatty acids is related to the reduction of breast cancer risk. In animal studies, α-linolenic acids have been shown to be able to suppress growth, size, and proliferation of cancer cells and also to promote breast cancer cell death. Other animal studies found that the intake of flaxseed combined with tamoxifen can reduce tumor size to a greater extent than taking tamoxifen alone. Additionally, some clinical trials showed that flaxseed can have an important role in decreasing breast cancer risk, mainly in postmenopausal women. Further studies are needed, specifically clinical trials that may demonstrate the potential benefits of flaxseed in breast cancer.

## Introduction

Cancer is one of the most serious health problems in Public Health given its high and increasing prevalence worldwide, being one of the main causes of morbidity and mortality and also responsible for a significant decrease in life quality. According to the World Health Organization (WHO), in 2012, 14 million new cases of cancer were diagnosed, which were responsible for 8.2 million deaths worldwide, with 521,000 of them being attributed to breast cancer (1–3).

In 2012, it was estimated that approximately 32.5 million people were cancer survivors, 5 years after being diagnosed with the disease. In 2030, it is expected that approximately 23.6 million new cases of cancer will be diagnosed each year (4).

In 2014, in Portugal approximately 27,200 people died of cancer, of which 16,600 were men and 10,600 were women (2).

According to the WHO, approximately one-third of the deaths caused by cancer are due to bad eating habits and lack of physical activity. By improving eating habits and increasing physical activity, more than 30% of the cancers diagnosed could be avoided. Thus, with the increased risk, a proper nutrition intervention is necessary (1–3).

Nutrition plays a fundamental role in cancer, as it can reduce complications that happen during treatment and can contribute to the patient’s well-being (3, 5).

Many people with cancer choose to make some changes in their eating habits while subjected to conventional, like chemotherapy. The patients do this in the hopes of decreasing the treatment’s severe side effects such as anxiety, depression, insomnia, headaches, nausea, and vomiting (among others) (6–9).

These less-conventional treatments may include diets with food that has proper nutritional characteristics to help fight the disease. Flaxseed has been one of the most studied foods regarding the possible relation to breast cancer. Some experimental studies in animals have been done but few progressed to clinical trials.

## Materials and Methods

To review the effect flaxseed may have in breast cancer, we conducted a bibliography research using sources from *PubMed*, and websites of institutions like Cancer Research UK and the WHO. The keywords used in the research were as follows: *cancer, flaxseed, lignan, breast cancer*, and *nutrition*.

## Results

### Breast Cancer

Breast cancer is considered as one of the most common cancers with the highest number of deaths worldwide. According to the WHO, it was estimated that in 2012, more than 1.68 million of women were diagnosed with breast cancer worldwide. Of these, approximately 521,000 died and in Europe, during the same year, more than 464,000 new cases were diagnosed and approximately 131,000 women died (1, 10, 11).

In 2014, in the United Kingdom, there were approximately 55,200 new cases of breast cancer (390 men and about 54,800 women), of which approximately 150 were diagnosed daily with approximately 11,400 deaths due to this kind of cancer. It is considered that one in eight women will be diagnosed with breast cancer during their lifetime (11).

In 2014, in Portugal, approximately 27,200 people died of cancer, with 1,791 of the victims being women with breast cancer. Also, approximately 6,088 new cases of breast cancer were diagnosed in women (2).

There are several factors that can be associated with breast cancer, such as gender, bad eating habits and respective lifestyles, family history, alcohol or tobacco consumption, lack of breast feeding, hormone treatments, overweight, and obesity, among others (10, 11).

After being diagnosed, many patients with breast cancer decide to change their eating habits and respective lifestyles (12, 13).

### Flaxseed and the Lignans

Flax (*Linum usitatissimum*), also known as linseed, belongs to the *Linaceae* family which originates from Europe, Asia, and the Mediterranean region. Flaxseed can be divided in two species: brown and golden. Golden flax develops in very cold climates, while brown flax develops in warmer and more humid climates. The latter must be ground to be better digested and absorbed by the body, thus increasing the bioavailability of the nutrients. It is considered a functional food that has nutrients with specific properties (antioxidant and/or antitumorigenic functions), such as omega-3 fatty acids, α-linolenic acid (LA), lignan, or fibers that are beneficial to one’s health, preventing some diseases, such as cancer and cardiovascular diseases, among others (14–16). Flaxseeds are rich in fiber and are suggested for situations of constipation, as they help to improve the intestinal function. They have omega-3 fatty acids that promote the reduction of cholesterol levels, thus preventing cardiovascular diseases. Additionally, they are still a good source of magnesium, phosphorus, manganese, vitamin B1, selenium, and zinc.

Although they are defined as one of the richest plant sources in omega-3 fatty acids, these seeds are also characterized by their lignan content. Although lignans are found in a variety of vegetable sources, such as whole grains, sesame seeds, vegetables, and fruits, flaxseeds have approximately 100 times more lignans than other foods (9, 17, 18).

Lignans are phytoestrogens that relieve the symptoms of menopause and can balance the effects of estrogen in the body by connecting to their receptors, as they have a very similar chemical structure as to an estrogen molecule (19).

**Figure 1:**
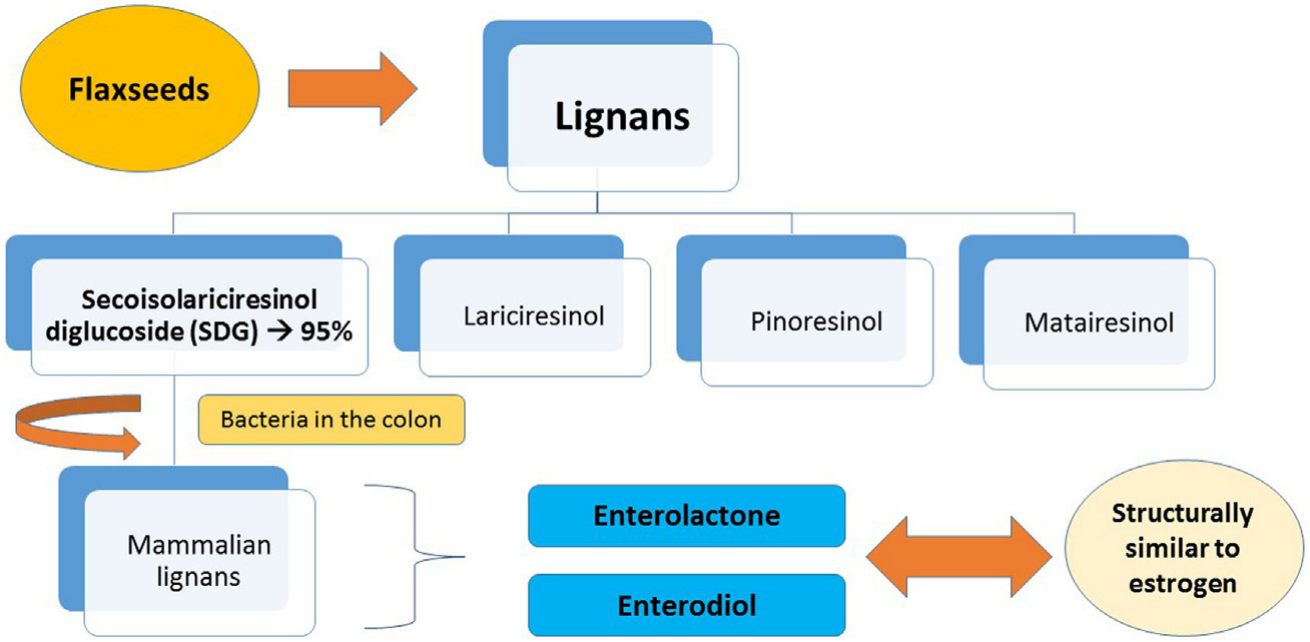
Metabolism of lignans in flaxseeds.

The predominant lignan in flaxseeds is secoisolariciresinol diglucoside (SDG), making up around 95% of the seed’s lignan content. The remaining 5% consist of lariciresinol, pinoresinol, and matairesinol. After SDG lignan ingestion, bacteria in the colon act by converting the lignan into mammalian lignans, enterolactone, and enterodiol. These are structurally similar to estrogen, and have antioxidant activity and a weak estrogenic action (Figure 1). It also works as an antiestrogenic because its structure is very similar to the main form of estrogen, which allows its binding to the cell’s receptors, thus inhibiting the growth of cancer cells (9, 20–23).

Breast tumors that contain estrogen receptors are called estrogen receptor positive (ER+) and tumors that lack estrogen receptors are estrogen receptor negative (ER−). Women who have ER+ tumors are more likely to respond to hormonal treatments than women with ER− tumors (24).

**Figure 2:**
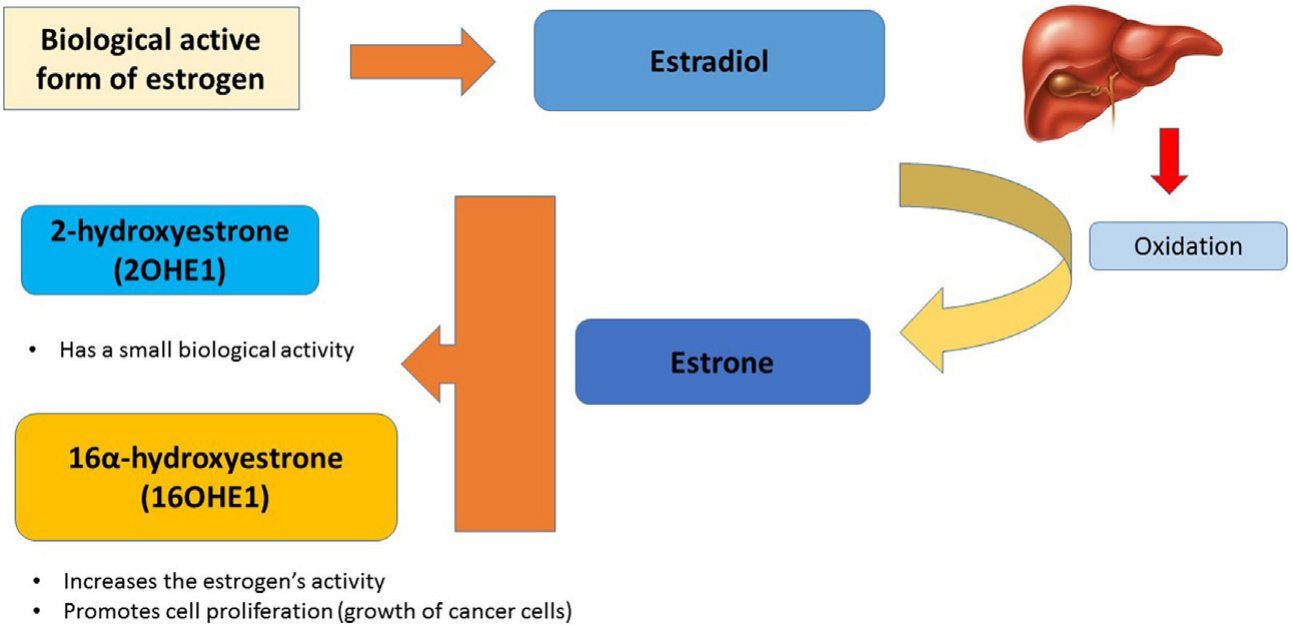
Influence of estrogen metabolism in cancer cells.

In our body, the biological active form of estrogen is estradiol, which is oxidized mainly in the liver to estrone. Estrone can be converted to two metabolites with different biological effects: 2-hydroxyestrone (2OHE1) and 16α-hydroxyestrone (16OHE1). While the first one has a small biological activity, the latter will increase the estrogen’s activity, promoting cell proliferation (growth of cancer cells) (Figure 2) (25, 26). Women who produce more 16OHE1 are likely to have an increased risk of breast cancer (27).

Two clinical trials concluded that 28 postmenopausal women, who followed a diet including 10 or 25 g of ground flaxseed for 7 or 16 weeks, witnessed an increased level of excretion of 2OHE1 in their urine, without an increase in the excretion of 16OHE1. These studies imply that flaxseed can have some protective effects in postmenopausal women (28).

### Omega-3 Fatty Acids and Breast Cancer

Polyunsaturated fatty acids (PUFAs) can be composed of omega-3 and omega-6. Linolenic acid and arachidonic acid (AA) are the main components of omega-6. The α-linolenic acid (ALA) is the precursor of the PUFA omega-3 family which forms eicosapentaenoic acid (EPA) and docosahexaenoic acid (DHA) (29, 30).

Omega-3 and ALA are also constituents of flaxseed. Flaxseed is considered as the best plant source of the essential omega-3 fatty acid. Studies suggest that the omega-3 fatty acid may have anticancer properties while omega-6 fatty acid can contribute to the development of cancer. Currently, in a regular diet, there is a higher amount of omega-3, than omega-6 (29, 30). Studies have revealed that PUFA omega-3 ingestion is associated with the reduction of risk of breast cancer (31). In animal studies, the ALAs have been shown to be able to suppress the growth, size, and proliferation of cancer cells. An increase in the death of these cells has also been observed (32, 33).

### Tamoxifen and Breast Cancer

Tamoxifen is a drug used in the treatment of breast cancer, mainly in women with ER+. It can be used as an adjuvant therapy for breast cancer or metastatic breast cancer. It is a type of hormone therapy that is done during 5–10 years with several side effects (34–37).

This drug works as an antiestrogen since, as it is an ER+ type of cancer, the tamoxifen acts throughout the whole body, blocking the action of estrogen on tumor cells, thus preventing their growth (34, 35).

According to experimental studies, flaxseed does not have any type of interaction with drugs used in breast cancer treatments and can provide an additional protective effect when consumed along with the treatment. In animal studies, it was also verified that flaxseed, flax oil, or lignan SDG ingestion, in combination with tamoxifen, reduced the tumor’s size to a greater extent than tamoxifen treatment alone. Until now, there are no clinical trials that can prove the benefits that flaxseed ingestion can have in women with breast cancer during tamoxifen therapy (38).

A study undertaken at the University of Toronto evaluated the effect of flaxseed and tamoxifen, alone and in combination, on the growth of ER+ human breast tumor cells in mice. These mice were injected with MCF-7 tumors and fed with different diets. The diets had 20–25 g of ground flaxseed, a tamoxifen pill (5 mg) or both. Tumor growth was monitored weekly. As a result, the flaxseed diet fed to the mice was the one that inhibited the growth of the ER+ human breast tumor cells. At low 17 beta-estradiol levels, flax inhibited tumor size approximately 74%, while at high 17 beta-estradiol levels it inhibited approximately to 22%. Furthermore, the combination of flax and tamoxifen inhibited tumor size more than 53%, as compared with tamoxifen action alone (39).

Other experimental studies conducted in mice injected with ER+ human breast tumor cells reveal that both at low- and high-estrogen levels (pre- and postmenopausal breast cancer), flaxseed either increased or kept tamoxifen’s effectiveness in decreasing tumor growth, cell proliferation, and increased apoptosis (38–42).

There are currently no known results from clinical trials regarding flaxseed ingestion during tamoxifen therapy (38).

### Animal Studies

In animal studies with mice injected with breast tumor cells, feeding them with flaxseed caused a decrease in tumor incidence, number, and size. These results were also revealed to be independent of the tumor’s stage (23).

A research group from the University of Toronto has also demonstrated that ground flaxseed has an effective anticancer activity. Their experimental study was conducted in mice to which tumors were administered, along with the introduction in their diet of a mixture of lignan. The result was a decrease in the tumor load due to the presence of flaxseed and lignan SDG in the mice diet (43–45).

Recently, the same research group injected another mice group with human breast tumor cells. While the cancer was progressing, the mice were on a regular diet for 8 weeks after cancer cells’ injection. One group was fed with 10% of flaxseed, while the other group kept the same kind of diet. The rate of the tumor growth was reduced by 45% due to flaxseeds (43–45).

In several other experimental studies, diets including 5 or 10% of flaxseed (approximately 25–30 g of flaxseed daily, in humans) inhibited the growth of the ER+ in human breast cancer cells injected in mice (39–42, 46, 47). The same happened with the growth of the ER− (44, 48, 49). Flaxseed also reduced metastasis of ER− breast tumor (44, 48, 50).

### Clinical Trials

Observational studies indicate that flaxseed consumption (approximately 32 g/daily) can reduce breast cancer risk (38, 51, 52). Lignans also contribute to the decrease of breast cancer risk. Vegetarians have a higher level of lignan ingestion, meaning that their breast cancer risk is lower than that of omnivores (53).

A study revealed that 70% of newly diagnosed patients with breast cancer consume food rich in lignans, 52% consume flax bread, and 30% consume flaxseed at least once a week (13).

A pilot study including 24 postmenopausal women with ER+ breast cancer was conducted to show the effects of flaxseed and the aromatase inhibitor, anastrozole (drug used in the treatment of breast cancer), and possible interactions between them in selected breast tumor characteristics and serum steroid hormone. These women were then divided randomly into four groups: Group 1 with 25 g/daily of ground flaxseed and 1 placebo pill daily; Group 2 with 1 mg/daily of anastrozole; Group 3 with 25 g/daily of ground flaxseed and 1 mg/daily of anastrozole; and Group 4 with 1 placebo pill daily. This study did not show any effects regarding flaxseed on the aromatase inhibitor activity in selected breast tumor characteristics and serum steroid hormone levels (9). Further studies are needed to support a possible interaction between flaxseed intake and the aromatase inhibitor––anastrozole.

Ingestion of flaxseed or bread containing this ingredient is associated with a 20% reduction in the risk of breast cancer, in accordance with the protective effect observed in lignans from other vegetables. This risk decrease may be related to a reduction in inflammation, since the presence of large amounts of lignans can lead to a decrease in several inflammatory markers (52, 54).

In two meta-analysis studies, it was found that a higher intake of lignans from dietary sources was associated with a significant reduction in postmenopausal breast cancer risk (54, 55). In a case-control study, the highest lignan consumption was associated with significantly lower postmenopausal breast cancer mortality but that association did not happen relatively to premenopausal breast cancer mortality (56).

A case-control study using the Ontario cancer registry database consisted of a random sample of women diagnosed with breast cancer, with the aim of analyzing the phytoestrogen intake (isoflavones and lignans) and their association with breast cancer risk. A food-frequency questionnaire was used, which also included foods rich in phytoestrogens. Lignan intake was associated with a reduction in the risk of breast cancer for all women, although this was only statistically significant in overweight women (BMI > 25). In premenopausal women, the total phytoestrogen intake was associated with a significant reduction in the risk of breast cancer, but only in overweight women. There was no association between breast cancer risk and the intake of phytoestrogen in postmenopausal women (51).

Another case-control study also using the Ontario cancer registry database conducted a food-frequency questionnaire with the aim of establishing if phytoestrogen intake during adolescence could protect against breast cancer in adulthood. The results of this study revealed that a higher phytoestrogen intake (isoflavones and lignans) during adolescence can be associated with a reduced breast cancer risk (57).

To explore the association between flaxseed ingestion and breast cancer risk, a case-control study was conducted by applying a food-frequency questionnaire to women who joined the *Ontario Women’s Diet and Health Study* in Canada (2002–2003). Both the monthly and weekly/daily consumption of flaxseed (approximately 32.5 g) and flax bread (1 unit, roughly 2.5 − 5 g of flaxseed) were associated with a significant reduction from 18 to 24% in the risk of breast cancer in all women. It was also showed that flaxseed only reduced breast cancer risk in postmenopausal women, while flax bread reduced breast cancer risk in both postmenopausal and premenopausal women (52).

A prospective cohort study, including 58,049 postmenopausal French women, found that those with the highest lignan ingestion (>1,395 μg/day) had a significantly decreased risk of breast cancer. The beneficial effects of lignans in this study were limited to ER+ breast cancer and progesterone receptor positive (58).

On the other hand, researchers conducted a double-blind, randomized clinical trial, with a placebo control in patients with breast cancer. The investigators tracked postmenopausal women that had been recently diagnosed with breast cancer and with a scheduled surgery. These women were then divided, randomly, into two groups: Group 1, which included 19 women, ate a muffin daily with approximately 25 g of powdered flaxseed, while Group 2, which included 13 women, ate a similar looking muffin, without flaxseed. Biopsies were performed in both groups, at the beginning of the trial, which were later compared with the pathology of the tumor removed during surgery, approximately 5 weeks after the study began. Women who ate the muffin containing flaxseed presented, on average, a decrease in the tumor cell proliferation, a reduction in the expression of c-erB2 levels (also known as HER2––an oncogene associated with the development and progression of breast cancer) and an increase in cell apoptosis. Researchers concluded that flaxseed has the potential of reducing tumor growth in patients with this type of cancer (38, 59).

In another clinical trial, researchers selected about 45 premenopausal women with a high risk of developing breast cancer (either with suspicious breast biopsies or former breast cancer survivors) and they gave them 50 mg of SDG lignan daily, or the equivalent of two spoons of powdered flaxseed. The researchers conducted needle biopsies on the breast tissue, both before and after the study, which lasted a year. Results showed that, on average, women had less breast precancerous changes after a 1 year period of taking daily flaxseed lignans, than they had before they started being studied and also that 80% (36 out of 45) had a decrease in the Ki-67 levels––a biomarker that signals the increase of cell proliferation. According to this clinical trial, SDG lignan can reduce the risk of breast cancer (60).

Some studies showed no association between risk of breast cancer and serum enterolactone levels (54, 55, 61, 62). There is a study that indicates a decreased breast cancer mortality with higher serum enterolactone levels (63). In a meta-analysis study, it was found that enterolactone biomarkers were associated with a statistically significant reduction of 28% in risk of postmenopausal breast cancer (64). A study including 1,140 postmenopausal patients with breast cancer showed that serum enterolactone concentrations in the highest quartile were associated with a reduction of approximately 40% in mortality (65). Also, a case-control study concluded that a greater amount of serum enterolactone levels can be associated with a decreased breast cancer risk (66). It is necessary to conduct more studies to be able to confirm if there is an association between serum enterolactone levels and breast cancer risk.

Five studies published between 2010 and 2011, which included patients diagnosed with breast cancer who were observed for a period of 6–10 years, with the aim of finding out if lignans could prolong the survival of patients with breast cancer. Through the measurement of food records or of serum lignan levels, researchers concluded that there was an increased exposure to lignans, which led to a significant reduction of approximately 40–53% in mortality and a 33–70% reduction in mortality by breast cancer. This increased exposure to lignans was mostly observed in postmenopausal women. There is a possibility that lignans could prolong the lifespan of patients with breast cancer, but further studies are needed to confirm this (15, 56).

Breast cancer survivors who have higher levels of lignans in their bloodstream and on their diet seem to survive for a significantly longer period of time (56, 65).

## Discussion

Since breast cancer has been considered one of the most common cancers with the highest mortality rate worldwide, it is important to include nutrition as part of this disease treatment. With the improvement in eating habits and the practice of physical activity, more than 30% of the cancers diagnosed could be avoided. It has been shown that an individualized nutritional intervention can reduce treatment complications and can improve the patients’ life quality. Flaxseed has been a vastly studied food due to the relation that it may have with breast cancer. Some experimental studies have been conducted in animals, but only a few clinical trials have been done in humans with the aim of discovering the effects of flaxseed on tumors and on the risk of this type of cancer.

Some studies revealed that the ingestion of omega-3 fatty acids is associated with the reduction of breast cancer. Animal studies showed that ALA can decrease the growth, size, and cell proliferation and can increase the death of breast tumor cells.

The majority of experimental studies conducted showed that flaxseed increases or maintains tamoxifen’s efficacy on the decrease of tumor growth on cell proliferation and on the increase of apoptosis. It is however necessary to conduct more clinical trials to confirm the association and respective efficacy of flaxseed with tamoxifen.

In several experimental studies, diets including 5 or 10% of flaxseed (approximately 25–30 g of flaxseed daily, in humans) inhibited the growth of the ER+ in human breast cancer cells injected in mice. The same happened with the growth of the ER−. Flaxseed also reduced the metastasis of ER− breast tumor.

During clinical trials, researchers have concluded that flaxseed has the potential to reduce the growth of tumors in patients with breast cancer, mainly postmenopausal women, and decrease the risk of this type of cancer.

Although many of the studies reported in this paper concluded that flaxseed intake may be related to the decreased risk of breast cancer and also to the reduction of the tumor’s growth and size, some studies including premenopausal and postmenopausal women did not show the same results.

However, more studies are still necessary, especially clinical trials, to verify the benefits of flaxseed on the treatment of breast cancer.

## Ethical Statement

None sought.

## Author Contributions

AC conceived the study, participated in its design and coordination, and drafted and authored the manuscript. PR participated in the study design, interpretation of the data, and helped to draft manuscript revisions. TS and PN were responsible for scientific writing and manuscript editing. All authors have read and approved the final manuscript.

## Conflict of Interest Statement

None of the authors reported any financial interests or potential conflicts of interest.
